# Development and Demonstration of a Method to Evaluate Bio-Sampling Strategies Using Building Simulation and Sample Planning Software

**DOI:** 10.6028/jres.115.008

**Published:** 2010-04-01

**Authors:** W. Stuart Dols, Andrew K. Persily, Jayne B. Morrow, Brett D. Matzke, Landon H. Sego, Lisa L. Nuffer, Brent A. Pulsipher

**Affiliations:** National Institute of Standards and Technology, Gaithersburg, MD 20899; Pacific Northwest National Laboratory, Richland, WA 99352

**Keywords:** agent, biological, modeling, planning, response, sampling

## Abstract

In an effort to validate and demonstrate response and recovery sampling approaches and technologies, the U.S. Department of Homeland Security (DHS), along with several other agencies, have simulated a biothreat agent release within a facility at Idaho National Laboratory (INL) on two separate occasions in the fall of 2007 and the fall of 2008. Because these events constitute only two realizations of many possible scenarios, increased understanding of sampling strategies can be obtained by virtually examining a wide variety of release and dispersion scenarios using computer simulations. This research effort demonstrates the use of two software tools, CONTAM, developed by the National Institute of Standards and Technology (NIST), and Visual Sample Plan (VSP), developed by Pacific Northwest National Laboratory (PNNL). The CONTAM modeling software was used to virtually contaminate a model of the INL test building under various release and dissemination scenarios as well as a range of building design and operation parameters. The results of these CONTAM simulations were then used to investigate the relevance and performance of various sampling strategies using VSP. One of the fundamental outcomes of this project was the demonstration of how CONTAM and VSP can be used together to effectively develop sampling plans to support the various stages of response to an airborne chemical, biological, radiological, or nuclear event. Following such an event (or prior to an event), incident details and the conceptual site model could be used to create an ensemble of CONTAM simulations which model contaminant dispersion within a building. These predictions could then be used to identify priority area zones within the building and then sampling designs and strategies could be developed based on those zones.

## 1. Introduction

The DHS Science and Technology Directorate (S&T) is tasked with researching and organizing the scientific, engineering, and technological resources of the United States, leveraging these existing resources into technological tools to help protect the homeland and supporting technological tools with standards. Standards development is critical to the deployment and performance evaluation of detection technologies, but the ability to detect contamination is equally dependent on the ability to find and recover contamination through sampling.

In the event of a bio-terror attack like the contaminated letter incidents of 2001, a building is first sampled to assess the extent of contamination and determine the appropriate decontamination method. Effective surface decontamination is critical to reducing the risk of physical transport and reaerosolization of contaminant materials. Post-decontamination sampling is used to determine efficacy of the decontamination effort and to assess the ability to clear the building of any remaining contamination. In order to increase the confidence in our ability to detect potential biological contamination events, the performance of sampling strategies and uncertainties associated with sampling methodologies must be characterized. In response to a 2005 report written by the Government Accountability Office (GAO-05-251) DHS organized a working group, the Validated Sampling Plan Working Group (VSPWG), composed of multiple federal agencies and national laboratories to ensure that the overall process of sampling activities has been validated. In response to the VSPWG efforts to develop performance characteristics for surface sampling methodologies, agency efforts include evaluating the impact of methodology limitations and sampling locations on the overall confidence of detecting surface contamination. The generation of a sampling plan based on probabilistic methods to choose the sampling locations provides a level of confidence in the overall ability to detect low amounts of surface contamination.

Over the past two years, a facility at Idaho National Laboratory served as the location for a multi-agency effort to experimentally evaluate the performance of sampling strategies to characterize and clear a building after a bio-contamination event. Both time and cost are significant constraints in large-scale field tests such as those conducted in Idaho. Modeling provides a means to expand our understanding of the distribution and transport of biological agents in a building beyond what can be explored by the physical and resource limitations of experimental testing. Model parameters can be adjusted to study the impact of variations in physical parameters that cannot be addressed experimentally in either a practical or economic manner.

The purpose of this project was to integrate and coordinate the use of CONTAM [2], an airflow and contaminant dispersal simulation program, with the Visual Sample Plan (VSP) [3] computer program to provide individuals and agencies responding to a biological contamination event with a software-based approach to generating sample plans. The objectives of this project were to demonstrate the process of using CONTAM simulations to develop zoning strategies and sampling designs in VSP and to explore whether there are consistent patterns of contaminant dispersion that would help in pre-planning zoning strategies within the building. Bio-threat release scenarios were generated for a single office building of moderate complexity using the CONTAM program (developed by and maintained at NIST). Release amounts, locations and circumstances including outdoor weather, ventilation system operation and other relevant building parameters were utilized to generate nearly four hundred release scenarios. Results of the NIST generated bio-threat dispersion and deposition scenarios were imported into the VSP software. VSP was developed at the Pacific Northwest National Laboratory (PNNL) to generate sampling plans that can include probabilistic sample site determinations and user selected judgmental sampling locations. PNNL visually analyzed the simulation results generated by NIST with CONTAM, looking for patterns, gradients and uncontaminated areas. In order to simplify the task of analyzing so many different scenarios, PNNL then performed a cluster analysis to group together scenarios that resulted in similar contamination patterns. Incident details, facility layout and CONTAM results were then utilized to classify the building spaces into four zoning categories according to their likelihood of being contaminated. The zoning procedure utilized the framework established by the Environmental Protection Agency (EPA) and Centers for Disease Control (CDC) through the VSPWG [4] to classify an area as a range from definitely contaminated to highly unlikely to be contaminated. Sampling approaches were generated for each zone classification.

Sampling plans generated utilizing CONTAM and VSP may provide individuals and agencies responding to a biological contamination event with a tool for evaluating the effects of building parameters, explore zoning strategies that cover most scenarios, and establishing sampling strategies for various scenarios. The computer tools can provide a means of assessing sampling strategies generated under different release scenarios prior to an actual contamination event and can aide in pre-planning and response plan generation by the first responder community for a biological attack.

A discussion of this effort, as well as a detailed description of the CONTAM and VSP tools, are provided in Sec. 2. The INL facility and the development of the CONTAM simulations are discussed in Sec. 3. Detailed results which address each of the three research objectives are presented in Sec. 4. Conclusions are given in Sec. 5.

## 2. Description of Modeling Tools

Two main software tools were employed in this project: CONTAM [2] and Visual Sampling Plan (VSP) [3]. CONTAM was used to simulate a set of contaminant release scenarios, and VSP was used to perform the statistical analysis of the simulation results. CONTAM provided results in the form of mass of contaminant deposited within each room of the simulated building. A third program was developed to convert the CONTAM results into a format that VSP could import. The following sub-sections describe each of these tools in more detail, with specifics on how these tools were used in this project provided in Section 3.

### 2.1 CONTAM

CONTAM is a computer program that is used to simulate building airflow and contaminant transport on a whole-building scale. The program was developed by the National Institute of Standards and Technology (NIST). CONTAM is referred to as a multi-zone or nodal network model, because the air volumes that make up a CONTAM representation of a building are treated as a set of nodes interconnected by airflow links. These nodes or zones are characterized by a single pressure, temperature and contaminant concentration, though pressure does vary hydrostatically within each zone.

For the purposes of analysis, a building is idealized by subdividing it into a set of zones, typically including rooms, major plenums between floors, and inter-floor shafts and chases. Zones are then interconnected by both intentional airflow paths, e.g., doors, windows, louvers and unintentional paths such as cracks that occur along the junctions between building partitions. Airflows occur between zones due to pressures arising from various driving forces including wind, temperature differences (stack effect) and mechanical systems. CONTAM allows the definition of mechanical ventilation systems either via a simplified representation of an air handling system or a fully detailed duct model. Contaminant concentrations within the building zones are affected by the location and strength of contaminant sources and removal mechanisms, e.g., material sinks and filters, as well as intra- and inter-zonal airflows. Detailed descriptions of CONTAM and multi-zone models in general, are available from several references [5–7] and the NISTwebsite [8].

One important aspect of CONTAM that is pertinent to this project is the contaminant element used to simulate the deposition of the biological agent. Particle deposition is typically characterized by a deposition velocity [9], and CONTAM provides the ability to model contaminant removal by deposition. The CONTAM deposition velocity model determines the mass of contaminant deposited on a given surface during a simulation based on the following equation:

$$M_{d}=\int_{t_{1}}^{t_{2}}\rho_{air}v_{d}A_{s}C(t)dt$$

*M_d_* = mass deposited [kg]
*ρ*_air_ = density of air [kg/m^3^]
*v_d_* = deposition velocity [m/s]
*A_S_* = deposition surface area [m^2^]
*C*(*t*)= mass fraction of contaminant [kg/kg]
*t* = time [s], (integration period was the same for all simulations)

CONTAM allows the definition of as many deposition surfaces in a zone as desired, using different deposition velocities for each as appropriate. However, all deposition surfaces must be placed within a CONTAM zone. This means that each of the deposition surfaces will see the same zone concentration, represented by *C*(*t*) in the previous equation, within each zone.

### 2.2 VSP

VSP is a software tool that employs statistically defensible approaches in developing sampling designs and analyzing data. It was developed by the Pacific Northwest National Laboratory to support the Data Quality Objectives (DQO) process [10]. It ensures the right type, quality, and quantity of data are gathered to support confident decisions and helps in evaluating tradeoffs between increased confidence in decisions and costs or number of samples required.

VSP allows the user to draw or import floor plans of buildings and generate and apply sampling designs to those floor plans. Sampling can be applied to the floor of a room or set of rooms, but can also be expanded to a 3D representation to include walls, ceilings, and furniture. Sample types (swab, wipe, vacuum) and points of interest (HVAC system, release point, sampling zones, etc.) can be user-specified. Many statistical sampling designs are available including random, systematic, sequential, adaptive cluster, collaborative, stratified, transect, multi-increment, combined judgmental and random, rank set, and judgmental sampling. A number of statistical analysis modules are available to complement the various sampling approaches. Statistical evaluations of the data are provided with decision recommendations, and most modules provide an automated report in text form.

For a particular release scenario, each room may have its own sampling design or be combined with other rooms to form a zone (which is different from the definition of zone used in CONTAM). Sampling designs can be generated for each room or zone, and results can be imported and analyzed using built-in statistical tests, visual analysis, and geostatistical mapping.

VSP has been developed with support from the U.S. Department of Energy (DOE), EPA, the Department of Defense, the Department of Homeland Security (DHS), CDC, and the United Kingdom Atomic Weapons Establishment. It is freely available at http://vsp.pnl.gov. In addition to supporting sampling within buildings, VSP has many sampling design and statistical analysis modules focusing on soils, sediments, surface water, streams, groundwater, and unexploded ordnance sites. VSP is recommended by many regulators for defensible sampling design and statistical analysis.

### 2.3 CONtoVSP

CONTAM and VSP were developed independently of each other. Therefore, their inputs and outputs are not immediately compatible. A process was developed to convert the CONTAM results into a format that VSP could import. This process involved defining a cross-reference file format that would map the space definitions of VSP into the deposition surfaces of the CONTAM building representation, modifying CONTAM results files to include information needed to facilitate the mapping, and developing a computer program to perform the conversion and generate the input files for VSP. The conversion program is referred to as CONtoVSP.

Typically, the VSP representation of a building surface would be subdivided into a set of grid cells that can take on numerical values, which in this case would be the mass deposited within the area of the cell. CONtoVSP obtains the mass deposited on each *deposition surface* from a CONTAM contaminant summary file (.csm) and apportions the total mass into a set of VSP grid cells that correspond to the *deposition surface* of CONTAM. The number of VSP cells associated with a room or sampling area is defined within VSP via the map file (.vsp). The number of cells that correspond to a CONTAM zone must also be set in the cross-reference file (.crf) in order for CONtoVSP to generate a properly formatted input file for VSP which requires a value for each sample cell defined in the VSP map file. This import file format can be viewed with VSP via the *View*—*Coordinates* menu command.

CONtoVSP enables the mass deposited on each CONTAM deposition surface to be distributed to the VSP grid cells either uniformly or randomly with a normal distribution. If the mass is to be distributed uniformly, then each cell will contain the same amount of mass, equal to the total mass deposited on the CONTAM deposition surface divided by the number of cells. If distributed normally, then a random set of values will be generated using a normal probability distribution function with a mean and standard deviation which are specified in the cross-reference file. CONtoVSP prevents negative values from being generated and normalizes the results by the total mass deposited on each CONTAM *deposition surface*.

## 3. Contaminant Transport Simulation Using CONTAM

This section describes the details of the modeling effort. It includes a description of the building, the CONTAM model, and the scenarios that were modeled in this study.

### 3.1 Building Description

**Fig. 1:**
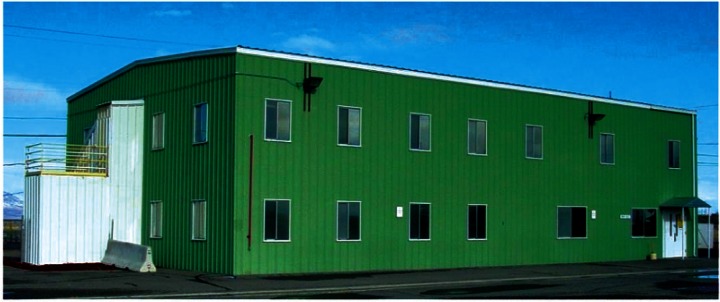
INL Building PBF-632.

**Fig. 2:**
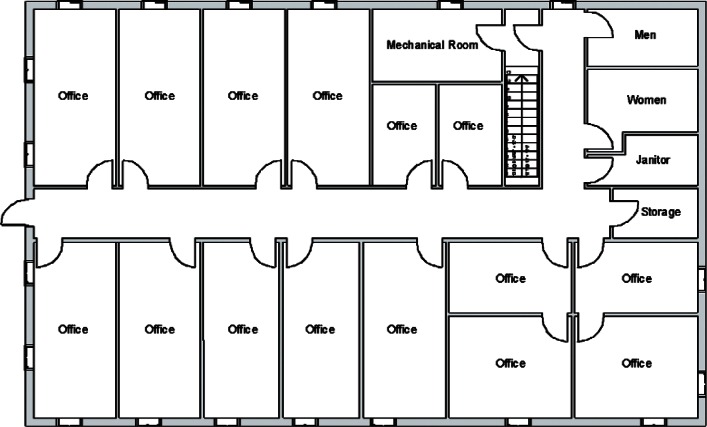
2nd Floor Plan.

**Fig. 3:**
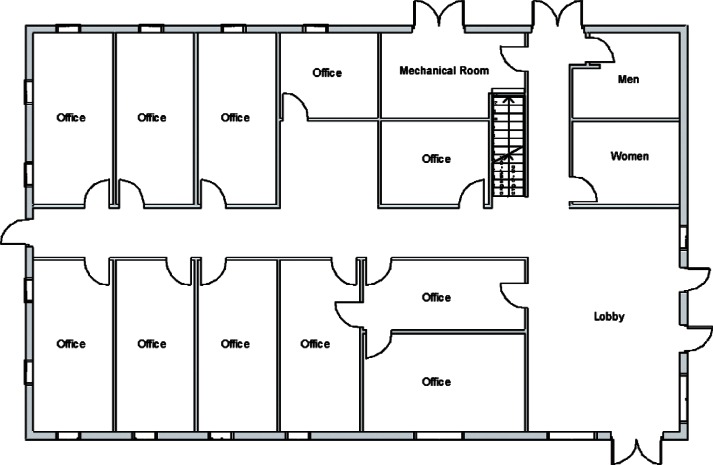
1st Floor Plan.

The building used for this study is PBF-632 located at the Idaho National Laboratory (INL) and shown in Fig. 1. Floor plans for the two floors of PBF-632 are shown in Fig. 2 and Fig. 3. Each floor is approximately 24.4 m × 15.2 m (80 ft × 50 ft) for a total of 372 m^2^ (4 000 ft^2^) per floor. As designed, each floor contains a constant volume air handler located within a mechanical room of the floor it serves. Outdoor air is brought in through an intake duct on the return air side of each air handler. Supply air ducts are located above suspended ceilings on the floor which they serve, and the space above the suspended ceilings also serves as a return air plenum. Return ducts draw air out of these plenums from just above each of the two mechanical equipment rooms. Supply air is provided to all occupied spaces except the restrooms and janitorial closet. Two return air grilles are located in the ceilings along the main hallway of each floor. Dedicated exhaust fans serve the four restrooms (two on each floor) and each of the mechanical rooms. Total design supply airflows for the 1st and 2nd floors are 1,200 L/s (2,550 cfm) and 1,180 L/s (2,500 cfm) respectively. Design restroom exhaust flows are 94 L/s (200 cfm) for each restroom and 47 L/s (100 cfm) for each mechanical room. The design outdoor air intake rate is unavailable.

### 3.2 CONTAM Model

The CONTAM model of the building was based on the building as designed. Design information was obtained from mechanical system drawings and electronic floor plans (AutoCAD format files, i.e., dwg files). Initially, the dwg files were imported into a 3D architectural Computer Aided Design (CAD) program that was then used to convert the main building geometry into an IFC-compliant (Industry Foundation Class) file. This file was then converted, via an in-house IFC to CONTAM converter, to an initial version of a CONTAM project file (.prj file) that included the building zones (rooms), doors and windows. Once the basic file was obtained it was modified to include building envelope and inter-zone leakages, plenums and a full duct system. For the purposes of this study, no leakage measurements were performed, so the envelope and inter-zone leakage values were assumed based on data in the literature [11, 12]. It is important to note that the purpose of this model was not to represent the actual building as-built, but to provide a realistic virtual test bed within which multiple release scenarios and building configurations could easily be applied and upon which the sampling strategies could be exercised.

**Fig. 4:**
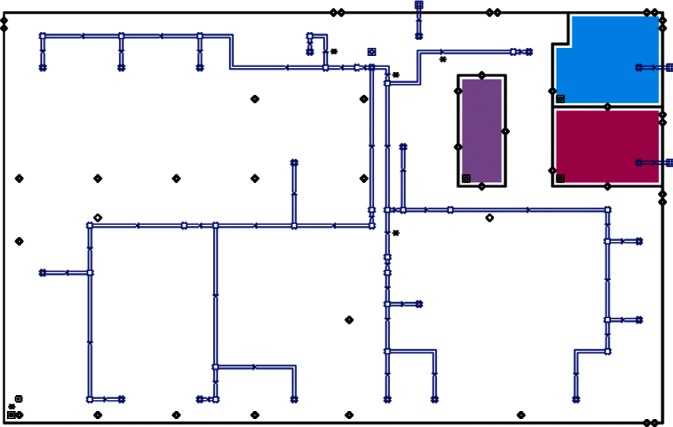
CONTAM representation of 1st Floor Plenum.

**Fig. 5:**
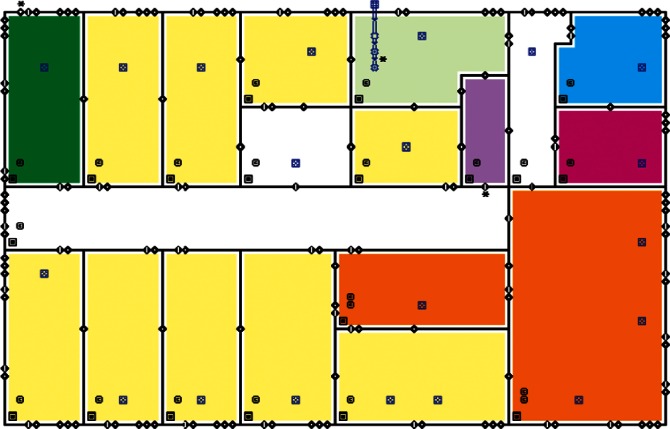
CONTAM representation of 1st Floor.

**Fig. 6:**
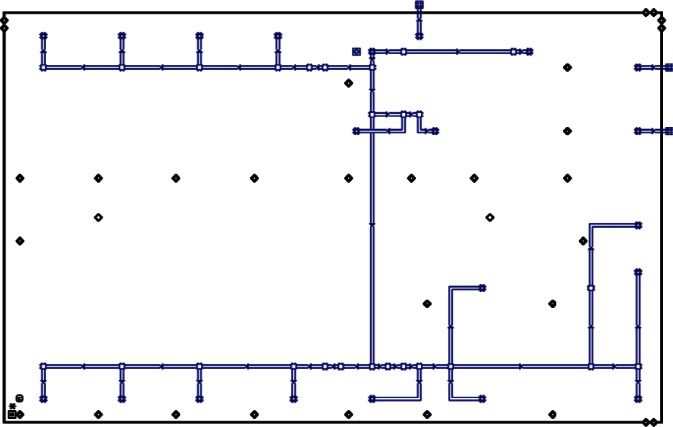
CONTAM representation of 2nd Floor Plenum.

**Fig. 7:**
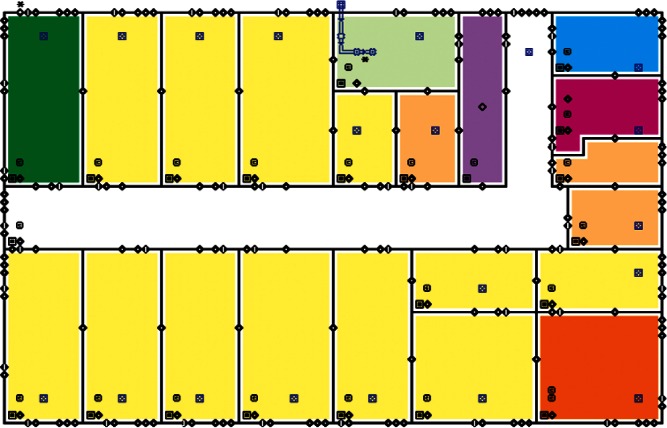
CONTAM representation of 2nd Floor.

Each level of the building is represented in CONTAM by a schematic of the floor plan via the CONTAM sketchpad. The model used in this study consists of four levels—the 1st and 2nd floors and their respective plenums that contain the ductwork. These four schematics are shown in Fig. 4, Fig. 5, Fig. 6 and Fig. 7. As seen in the figures, the building is represented by various icons which include zones, airflow paths between zones, ducts and the deposition surfaces. Zones are delineated by the solid black lines. Airflow paths are represented by the diamond-shaped icons located along the walls and on the floors of all but the bottom level. The double blue lines and icons represent the duct system. Each zone has a deposition surface icon associated with it. The CONTAM colors and icons are defined as follows:

**Figure:**
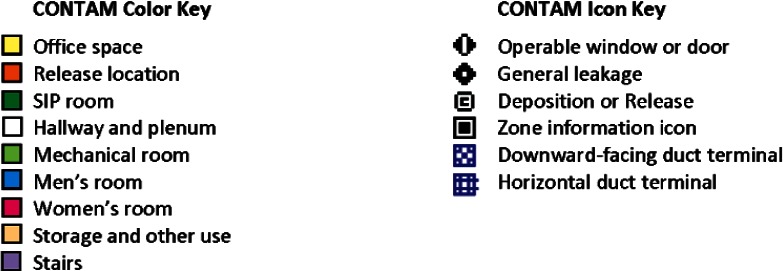


The release agent was modeled as a particle having an equivalent diameter of 1.06 μm. The deposition surfaces were assumed to consist of only the floor area of each zone. A constant deposition velocity of 3.78 × 10^−5^ m/s [13] was assumed for all deposition surfaces within the CONTAM model. These surfaces were subdivided into 61 cm × 61 cm (2 ft × 2 ft) grid cells within VSP.

### 3.3 Scenarios Modeled

The main benefit to using simulations to evaluate sampling strategies is the ability to perform multiple release scenarios under varying building operating and environmental conditions. CONTAM allows for releases to be simulated from within any of the building zones with varying amounts and time profiles. It also allows for releases external to the building by either targeting specific building openings or assuming a concentration profile around the building. CONTAM also allows the simulation of various building configurations including variations in door and window opening status, variations in fan on/off status and ventilation flow rates, as well as variations in weather conditions.

Table 1 provides a list of the factors that were considered in the scenarios simulated and their associated levels. This is by no means an exhaustive list of potential variables and variations, but it was considered to be the most ambitious set of scenarios allowed under the resources available for this project. This set of factors and levels yields a total combination of 384 scenarios. A project file generation tool was developed to create the 384 test cases and batch files were used to run CONTAM and the CONtoVSP conversion tool. In this way the full set of simulations could be performed more quickly and reliably.

The release zones are shown in orange in Fig. 5 and Fig. 7 (near the bottom-right corners of the schematics) and the zones having the dedicated fans are shown in dark green (top-left corners of the schematics). In the case of the outdoor release, the contaminant is simulated to be uniformly distributed around the building. All simulations were for 24 h periods with 1 min time steps. All releases were modeled to occur for 1 min starting 1 h into the simulation. When the dedicated outdoor air fan was turned on, the duct that served the zone into which the outdoor air was provided was closed off in order to prevent the agent from being re-circulated into the zone. Thus, when the release was from an interior source, these zones would serve as a form of shelter-in-place (SIP) zones and should have very little to no contamination. This was necessary to support one of the objectives of this research effort, i.e., the development of zoning strategies.

## 4. Analysis of Simulations and Sampling Strategies Using VSP

**Fig. 8:**
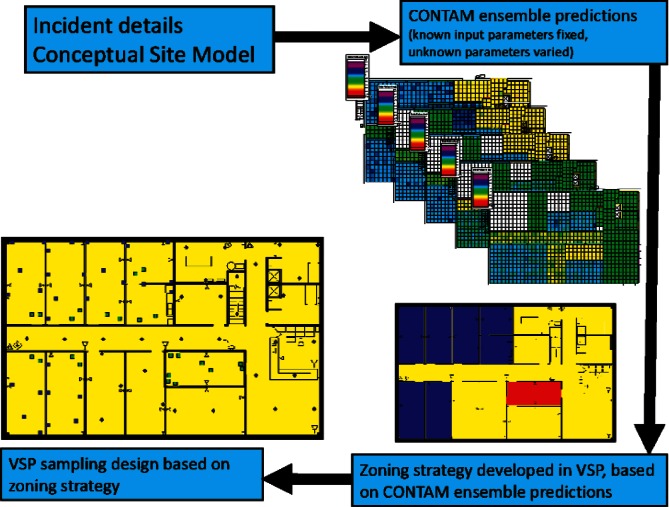
Illustration of CONTAM-VSP integrated sample design process.

One of the fundamental outcomes of this project was a demonstration of how CONTAM and VSP can be used together to effectively develop sampling plans to support the various stages of response to an airborne chemical, biological, radiological, or nuclear (CBRN) event. Following a CBRN event, incident details and the conceptual site model (CSM) could be used to create CONTAM predictions of contaminant dispersion within a building. These predictions would then be used to identify priority area zones within the building, and then sampling designs and strategies could be developed based on those zones. As discussed in Sec. 2.1 and 3.2, a sizeable number of input parameters must be specified in order to execute the CONTAM model. It is plausible that reasonable estimates could be made for some of the CONTAM parameters using the incident details and the CSM. However, some of the CONTAM input parameters will remain unknown or at least less well known. Consequently, a group (or ensemble) of CONTAM dispersion predictions could be made by holding constant the input parameters which are known and varying the input parameters that are unknown. Visual analysis of the ensemble of predictions could then be used to develop a priority zoning strategy within the building. Sampling plans, in turn, would be developed in VSP to match the sampling objectives and characteristics of each of the zones. In short, CONTAM predictions can be used to create more efficient sampling designs in VSP. The integrated CONTAM-VSP process is illustrated in Fig. 8.

PNNL used VSP to analyze a large number of simulated scenarios generated by CONTAM. Five zoning strategies were developed based on typical patterns observed in the CONTAM simulation results. For each zoning strategy, four sampling approaches were evaluated for the various scenarios: judgmental, purely probabilistic, purely probabilistic with zoning, and combined judgmental and random (CJR) with zoning.

Section 4.1 discusses the process of using visual and cluster analysis to explore whether there are consistent patterns of contaminant dispersion that would help in pre-planning the zoning strategies within the building. The definitions and characteristics of the various zones are presented in Sec. 4.2. The demonstration of how the results from Sec. 4.1 are used to develop zoning strategies is presented in Sec. 4.3. Section 4.4 presents comparisons of the performance of the various sampling approaches across five typical scenarios identified by the visual and cluster analysis shown in Sec. 4.1.

### 4.1 Identifying Patterns of Contaminant Dispersion

#### 4.1.1 Visual Analysis

The results of the 384 CONTAM simulations were converted by CONtoVSP (Sec. 2.3) into the file format used for VSP files. In this format, each floor has its own file containing the response values (contaminant levels) for each grid cell organized by rooms. Files were provided using both the uniform and normal distribution methods for distributing contaminant to grid cells within each room (Sec. 2.3). All numerical analyses used data from the uniform distribution method. The methods produced similar images when viewed in VSP, and either set of data (uniform or normal) could be used for visual analyses. However, data from the normal distribution method have more texture instead of giving rooms solid colors, and this makes visual comparisons of adjacent rooms somewhat easier. Therefore, all images in Sec. 4 of this report are from data using the normal distribution method. To better account for both linear and non-linear patterns on the floors, PNNL created a second set of files that use base 10 logarithms of the raw values, so that both the raw and log-scaled data could be analyzed. This was done for both floors in every simulation, resulting in four VSP files for each of a scenario's sets of data (uniform or normal), the raw and log of the 1st floor values, and raw and log of the 2nd floor values. Visual analysis was performed using the *Color By Values* feature of VSP [3]. This feature maps the data values in each cell to a continuous color scale, making it easy to visualize the location and intensity of contamination. A total of 3,072 (2 × 2 × 2 × 384) images were produced and analyzed visually with the objective of identifying scenarios that:

1. Showed contamination gradients or trends on individual floors.
2. Showed floors with little or no contamination.
3. Showed other interesting patterns including unique patterns isolated to only a handful of simulations, or scenarios that summarized a larger number of scenarios with similar dispersion of contaminant.

Using VSP’s menu commands to open and view each of the 3,072 files would clearly be a slow process. So, a “scenario viewer” was developed using a Python script to automate the process.

Scenarios are called up one-at-a-time, allowing the user to look at the 1st floor, and then the 2nd, by hitting a “Next” button. If a scenario fit one of the three categories, a “Save” button allowed the scenario to be stored for future use. If both files have been viewed, or a scenario was saved, the viewer proceeds to the next scenario. The raw and log values were analyzed in separate runs of the viewer for both the uniform and normally distributed sets of data.

Approximately 50 % of the scenarios fell into one of the three categories listed above. Examples of scenarios not included in this 50 % include cases in which most, if not all, of the rooms were contaminated with no discernible gradient, or contamination was uniform across all or most of the rooms.

#### 4.1.2 Cluster Analysis

The visual analysis revealed many different patterns across the different scenarios. The next step was to perform a numerical analysis of the data that would help put the results of the visual analysis into perspective. It was decided that a cluster analysis was well-suited for summarizing the numerical results.

We chose to use the rooms as the individual units for the cluster analysis. The numerical values used in the analysis were the average contamination levels of the grid cells in each room. This results in having the cluster analysis generally group together scenarios with similar levels of contamination in the same rooms so that scenarios with similar dispersion patterns and similar contaminant levels in rooms will tend to group together.

**Fig. 9:**
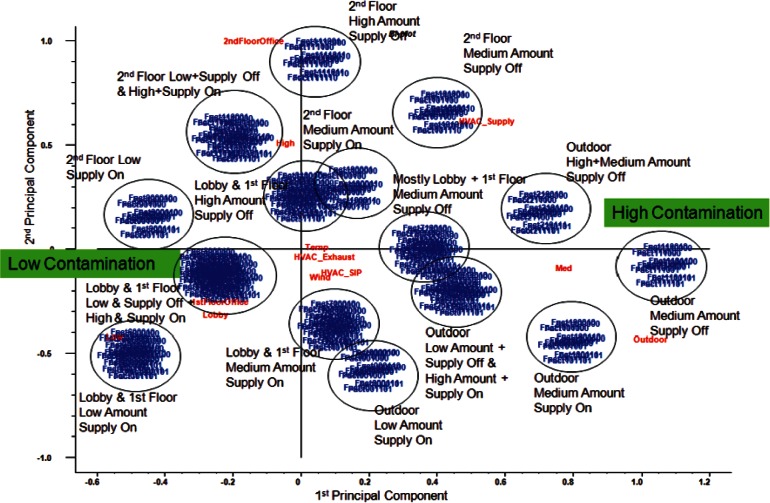
Partial Least Squares (PIS) Bi-Plot of the 384 CONTAM simulations.

Results of a partial least squares (PLS) regression [14] bi-plot are shown in Figure 9 generated using The Unscrambler software [15]. In PLS, each room has a variable stating the scenarios' levels of contamination, and these rooms are simultaneously modeled using the experimental variables previously outlined in Table 1. Unlike traditional regression methods, all rooms are modeled together taking into account their correlations and the data structure of both the contamination levels of the rooms and the experimental variables. Clusters of scenarios can be shown graphically with the regression variables overlayed on the plot to show which variables are positively or negatively correlated with a cluster. Variables on the outside of the plot and near a cluster will tend to be positively correlated with that cluster. Variables on the outside of the plot and far away from a cluster will tend to be negatively correlated with that cluster. In Fig. 9, clusters near the “High Contamination” are those with the highest levels of contamination, and likewise “Low Contamination” indicates clusters with lower levels of contamination.

PLS was used largely because in addition to identifying clusters, it also attempts to identify how the combinations of the various CONTAM factors may influence the way in which the scenarios are clustered. The results show approximately 15 distinguishable groups of scenarios. Groups were largely determined by three of the explanatory variables that were significant in the PLS model: release point, the amount of contaminant released, and whether the supply fans were on or off. In order to reduce the large number of CONTAM simulations into smaller groups for analysis, a 3 × 3 × 2 factorial structure was used to create 18 clusters by using release point (1st floor office and 1st floor lobby combined, 2nd floor, or outdoor), the amount of contaminant released (low, medium, or high), and supply fan status (on or off).

The resulting clusters of scenarios were defined as follows:

1. 1st floor release (lobby or office), low amount, supply fan on
2. 1st floor release (lobby or office), medium amount, supply fan on
3. 1st floor release (lobby or office), high amount, supply fan on
4. 1st floor release (lobby or office), low amount, supply fan off
5. 1st floor release (lobby or office), medium amount, supply fan off
6. 1st floor release (lobby or office), high amount, supply fan off
7. 2nd floor office release, low amount, supply fan on
8. 2nd floor office release, medium amount, supply fan on
9. 2nd floor office release, high amount, supply fan on
10. 2nd floor office release, low amount, supply fan off
11. 2nd floor office release, medium amount, supply fan off
12. 2nd floor office release, high amount, supply fan off
13. Outdoor release, low amount, supply fan on
14. Outdoor release, medium amount, supply fan on
15. Outdoor release, high amount, supply fan on
16. Outdoor release, low amount, supply fan off
17. Outdoor release, medium amount, supply fan off
18. Outdoor release, high amount, supply fan off.

Clusters involving 2nd floor and outdoor release points (7–18) each consisted of 16 CONTAM simulations. Clusters involving 1st floor release points contained 32 CONTAM simulations because the 1st floor office and 1st floor lobby release points were combined since they resulted in similar dispersion patterns and levels throughout most of the building.

The visual analysis of the clusters also revealed patterns regarding the variables used for choosing these initial clusters. First, the supply fan appeared to be a large factor in determining how much of the building was contaminated. When the supply fan was on, all or most of the building was contaminated. When the supply fan was off, often only a smaller portion of the building around the release point was contaminated.

The amount of contaminant released affected the amount of contaminant in each of the rooms, but did not appear to have a large effect on dispersion patterns. For instance, the simulations with the same factors other than having low, medium, or high amounts of contaminant tended to have the same rooms contaminated regardless of the amount of contaminant. This is to be expected, because the contaminant has no effect on airflow patterns and deposition rates as modeled by CONTAM.

#### 4.1.3 Visual Analysis of Clusters

Following the initial visual analysis and the cluster analysis, a new visual analysis of the scenarios in each individual cluster was then performed. In the original visual analysis of all 384 CONTAM simulations, it was learned that the log-scaled data was much more intuitive for finding patterns using VSP. Therefore, the remaining analysis was performed using only the log-scaled data. Also, in order to maintain consistency across scenarios during the visual analysis, the color scales were fixed in VSP as opposed to each scenario being auto-scaled based on its individual range of values. Images were produced of maps of the 1st and 2nd floors for each scenario and their levels of contamination shown by a color scale as in the original visual analysis. This time, color scales were kept fixed and the images were organized by cluster.

As a result of this analysis, the set of clusters were reduced even further from 18 down to 5. After examining images within each cluster, it became more apparent that the amount of contamination had very little influence on which rooms were contaminated, and therefore the amount of contaminant would not be a major factor when determining zoning strategies. Clusters with the same release point and supply fan setting were combined as were those with outdoor releases. For outdoor releases, it was obvious that a single zoning strategy was sufficient since the outdoor release scenario (a plume surrounding the building) always contaminated each room in the building to at least a small extent. This resulted in the following five clusters, which from this point forward will be referred to as Clusters 1–5:

1. 1st Floor (Office or Lobby) Release, Supply Fan OFF (96 CONTAM scenarios)
2. 1st Floor (Office or Lobby) Release, Supply Fan ON (96 CONTAM scenarios)
3. 2nd Floor Office Release, Supply Fan OFF (48 CONTAM scenarios)
4. 2nd Floor Office Release, Supply Fan ON (48 CONTAM scenarios)
5. Outdoor Release (96 CONTAM scenarios).

Images from one of the scenarios in each of Clusters 1–5 are shown in Fig. 10, Fig. 10, Fig. 11, Fig. 12, Fig. 13, Fig. 14, Fig. 15, Fig. 16, Fig. 17, Fig. 18 and Fig. 19 as examples of contaminant deposition patterns for the scenarios in each cluster.

### 4.2 Zone Classifications

The concept of dividing the building into classes is consistent with the Multi-Agency Radiation Survey and Site Investigation Manual (MARSSIM) developed by EPA, Nuclear Regulatory Commission (NRC), and DOE [16]. According to the MARSSIM, during the characterization phase of a release event, the rooms of the building should be divided into one of four possible priority areas or zones. These zones are not to be confused with CONTAM zones. The following is the set of classification criteria [17] into which the building areas were categorized for this study:

**Class 1 Zone:** Definitely Contaminated or Assumed to Be Contaminated (i.e., Extremely High Likelihood of Being Contaminated).
**Class 2 Zone:** High Likelihood of Being Contaminated.
**Class 3 Zone:** Low Likelihood of Being Contaminated.
**Class 4 Zone:** Extremely Low Likelihood of Being Contaminated [18].

Based on the scenarios analyzed, the building used in this case can be zoned into Class 1, 2, or 3 zones. A separate zoning strategy was developed for each of the five clusters of scenarios, and these will be shown later. The relatively small size of the building, both floor area and number of floors, as well as the tendency of the ventilation system to mix the air throughout each floor, leads to reduced expectancy for Class 4 zones. Therefore, all rooms would be classified as at least Class 3 zones. Class 4 zones are more likely to occur in situations where a release would be isolated to a relatively small area of a large facility, either due to the nature of the release (i.e., location, amount or mechanism), building configuration, or system operation.

The sampling approaches for each zone class are different because the sampling objectives and decision criteria can be based on the pre-event information developed for each part of the building through, for example, the visual and cluster analysis carried out herein. For example, during the characterization phase of a release event, Class 1 and Class 2 zones might warrant the use of a sampling approach that is geared towards determining the extent of contamination. While Class 3 or 4 zones can be handled with a clearance sampling approach where enough samples are taken to determine with high confidence that little or no contamination is present in the area, and decontamination may not be necessary. The recommended sampling strategies for each phase of the response and each zone are presented below.

#### 4.2.1 Class 1 Zones

Class 1 zones are known or assumed to be contaminated, such as the location of the release point of the contaminant, or rooms that would almost certainly be contaminated (provided the release point is known). These rooms will presumably need to be decontaminated. If desired, judgment samples can be taken using the sampling expert's best professional judgment, which may include choosing locations in rooms most likely to be contaminated and surfaces from which an accurate sample can be most easily extracted. The red area in Fig. 20 shows a Class 1 area, which in this case is the known release point. In some cases where a very large area is expected to be contaminated (usually in a much larger building), a hotspot sampling design may be applied to delineate areas of contamination.

#### 4.2.2 Class 2 Zones

Class 2 zones are assumed to have a high probability of being contaminated, but there is no obvious evidence that points to it conclusively being contaminated. Initially, judgmental samples may be taken in the most likely areas of contamination. In Fig. 20 the yellow areas are Class 2 Zones. These are rooms surrounding the suspected release point (the red Class 1 Zone). If unacceptable levels of contamination are present, then the area can be reclassified as a Class 1 zone and can be scheduled for decontamination. If judgmental samples are clean, then a more extensive statistical sampling design can be applied to determine a course of action for the zone. A hotspot sampling design could be implemented to delineate possible areas of contamination, or the initial assessment of the zone being Class 2 may be reconsidered. A clearance design might be applied to determine with statistical confidence that a high percentage of the area is free of contamination.

#### 4.2.3 Class 3 Zones

Class 3 zones are assumed to have a fairly low probability of being contaminated. In Fig. 20, the blue areas are Class 3 Zones. On the 1st floor these rooms are not directly adjacent to the room in which the release occurred and tended to be “upstream” from the source in terms of airflow direction for the cases simulated. On the 2nd floor, the Class 3 rooms show that most of the 2nd floor has a high probability of not being contaminated. The sampling objective for Class 3 zones is to take enough samples to either confidently conclude that the zone is not contaminated above the acceptable contamination level (ACL) or detect contamination if it exists. It is recommended that enough samples be taken to make a confidence statement about the zone. This statement is referred to as an X %/Y % upper percentile tolerance statement [19, 20] and is stated in the form “there is X % confidence that at least Y % of the surface area is uncontaminated (or below the ACL)”.

Two sampling strategies are recommended for sampling Class 3 zones: a purely probabilistic sampling approach and a combined judgmental and random (probabilistic) approach (CJR). In a purely probabilistic sampling approach, random samples are taken, all samples are expected to have an equal likelihood of being contaminated, and no prior assumption is made about the probability of the zone being contaminated. In the CJR approach [21], randomly located (probabilistic) samples are used to augment judgmental samples in order to make the desired X %/Y % confidence statement. In the CJR method, judgmental samples can be assumed to have a greater likelihood of containing detectable contamination than probabilistic samples. Also, before any sampling takes place, a Bayesian prior distribution is determined by the a priori probability that a judgmental sample will contain detectable contamination. This is helpful for situations in which experts feel there is some evidence that a zone may be clean, but they wish to take samples to achieve a high level of confidence. The CJR method, when appropriate, can be advantageous because, it requires fewer total samples than a purely probabilistic approach.

#### 4.2.4 Class 4 Zones

Class 4 zones have an extremely low likelihood of being contaminated. These are zones for which there is sufficient evidence to conclude that no contaminant could have entered them. In general, sampling is not required for these zones—however, clearance sampling may be required by stakeholders, in which case a CJR design would be recommended. If there is any non-negligible chance that an area could be contaminated, the zone should not be classified as Class 4.

### 4.3 Developing Zoning Strategies

The scenarios within each cluster tend to have similar patterns of dispersion. A visual analysis was conducted on each of the five clusters to determine contamination patterns on each floor and to determine which rooms tended to be contaminated either all of the time, most of the time, rarely, or never. These results were then used to classify rooms as Class 1, Class 2, Class 3, or Class 4 zones. The following sections describe the zoning process and strategy for each of the five clusters.

#### 4.3.1 Cluster 1 Zoning: 1st Floor Releases (Lobby and Office) with Supply Fans OFF

The zoning strategy for Cluster 1, (all scenarios with a 1st floor release with Supply fans OFF) is shown in Fig. 20. The strategy for Cluster 1 as illustrated in Fig. 20 reflects a 1st floor office release, but the cluster also contains 1st floor lobby release scenarios. These release points were combined into the same cluster because the spread of contamination tended to be very similar for the two different release points. Therefore, these scenarios were zoned the same with the exception that the 1st floor office and lobby were zoned as either Class 1 or Class 2 depending on which was the release point.

1st floor releases with Supply fans OFF resulted in the point of release always being contaminated (the red room in Figure 20). Therefore, the release room is always labeled Class 1. The surrounding rooms, hallway, and mechanical room and bathrooms were usually contaminated and are labeled Class 2 zones (yellow rooms). Offices on the opposite side of the floor (left end of building on floor plans) were occasionally contaminated and are Class 3 zones (blue rooms).

The 2nd floor was usually contaminated in the area above the mechanical room and bathrooms of the 1st floor. These are labeled Class 2 zones (yellow rooms in Fig. 20). The remaining rooms on the 2nd floor were occasionally contaminated and are labeled Class 3 zones (blue rooms).

#### 4.3.2 Cluster 2 Zoning: 1st Floor Releases (Lobby and Office) with Supply Fans ON

Figure 21 shows the zoning strategy for Cluster 2 (all scenarios with a 1st floor release and Supply Fans ON). On the 1st floor, the release point (office), a neighboring room, and the hallways were always contaminated and are Class 1 zones (red rooms in Fig. 1). The rest of the floor, with the exception of one corner office, was usually contaminated and classified as Class 2 zones (yellow rooms). The previously mentioned corner office was occasionally contaminated and classified as a Class 3 zone (blue room).

The entire 2nd floor was usually contaminated with the exception of one corner office (which corresponds to the Class 3 office on the 1” floor), and these rooms were classified as Class 2 zones (yellow rooms in Fig. 21). The previously mentioned corner office was classified as a Class 3 zone (blue room). These two corner offices on the 1St and 2nd floors labeled as Class 3 zones were the so-called SIP zones mentioned in Sec. 3.3.

#### 4.3.3 Cluster 3 Zoning: 2nd Floor Office Releases With Supply Fans OFF

Figure 22 shows the zoning strategy for Cluster 3 (all scenarios with a 2nd floor office release and Supply Fans OFF). On the 2nd floor, the release location and a neighboring office were always contaminated and are categorized as Class 1 zones (red rooms in Fig. 22). The other offices around the release point and on the right half of the floor were usually contaminated, and are categorized as Class 2 zones (yellow rooms). The remaining rooms on the other half of the building were only occasionally contaminated and categorized as Class 3 zones (blue rooms).

The entire 1st floor was usually not contaminated, and therefore the entire floor is categorized as Class 3 (the entire floor is blue in Fig. 22).

#### 4.3.4 Cluster 4 Zoning: 2nd Floor Office Releases With Supply Fans ON

Figure 23 shows the zoning strategy for Cluster 4 (all scenarios with a 2nd floor office release and Supply Fans ON). On the 2nd floor, the release location and a neighboring office were always contaminated and are categorized as Class 1 zones (red rooms in Fig. 23). Most of the other areas on the 2nd floor were usually contaminated and are categorized as Class 2 zones (yellow rooms). Two corner offices near the SIP room were occasionally contaminated and are categorized as Class 3 zones (blue rooms).

Most of the offices and areas on the 1st floor were usually contaminated and are categorized as Class 2 zones (yellow areas in Fig. 23). Four offices in one corner of the floor near the SIP room were occasionally contaminated and categorized as Class 3 zones (blue rooms).

#### 4.3.5 Cluster 5 Zoning: Outdoor Releases

Figure 24 shows the zoning strategy for Cluster 5 (all scenarios with an outdoor release). All rooms of both the 1st and 2nd floor were always contaminated. This was most likely due to the type of outdoor release simulated in this exercise, which was a plume passing over the building, and presumably contaminant making it into enough areas of the building that it spread to all parts either by ventilation system mixing or airflows induced by wind and stack effect. Since there is no release point within the building, all areas of the building were classified as Class 2 zones indicating contamination is highly likely.

### 4.4 Comparing Sampling Strategies

Building airflow and contaminant transport modeling can be used to compare different sampling strategies (as a supplement to experimental efforts). The single zone (uniform concentration) assumption of the multi-zone model resulted in entire rooms being contaminated. Therefore, hotspot designs are not applicable to these circumstances. Hotspot sampling designs are more suited to scenarios that result in portions of rooms being contaminated. While hotspot scenarios were not addressed in this study, CONTAM does provide the ability to simulate more detailed contaminant distribution on a sub-room scale, e.g., using one-dimensional convection/diffusion and computational fluid dynamics (CFD) within selected zones. Examples of the types of sampling strategies that could be deployed under the uniform concentration assumption of CONTAM include the following:

1. Purely Judgmental—The sampling team uses only their professional opinion to choose sampling locations.
2. Purely Probabilistic—Sample locations are chosen randomly or using a randomly placed grid.
3. Purely Probabilistic given a Zoning Approach —Depending on the sampling objective, either hotspot sampling is performed, or a number of samples are taken to make an X %/Y % confidence statement.
4. Combined Judgmental/Random (CJR) with Zoning—Both judgmental and probabilistic samples can be taken to make X %/Y % confidence statements. Judgment samples potentially carry more weight, and a Bayesian prior is applied that factors in existing, but not sufficient, evidence that the area may be clean. Random samples are also taken to supplement judgmental samples and to establish a sufficient number of samples to achieve the X %/Y % confidence statement.

The following example is presented to show how different sampling strategies can be compared. In this example, the four sampling strategies mentioned above are applied to Cluster 1. VSP was used to generate the probabilistic sample locations. Because entire rooms were contaminated in the methodology used, locations of judgment samples were not critical for this exercise and do not affect the results. Therefore, judgmental sample locations were selected by PNNL based loosely on locations from previous live tests [22]. However, during an actual contaminant release, judgment sample locations would be chosen by contamination sampling experts depending on the amount of information known about the release [4]. If judgment sample locations were to influence the results, such as scenarios where rooms were only partially contaminated, the assistance of a sampling expert should be used to choose judgment sample locations.

**Fig. 10:**
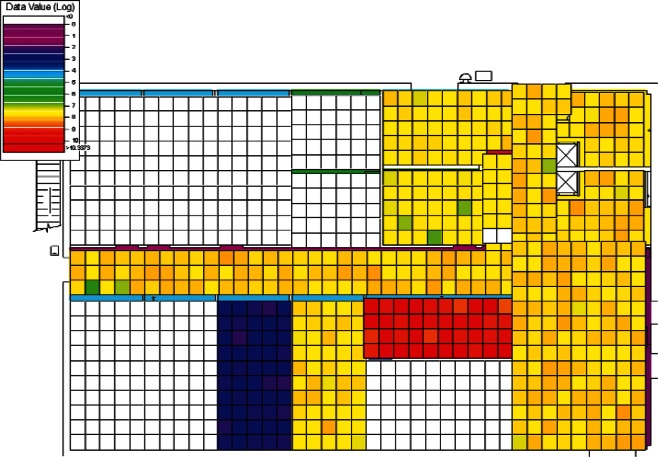
Cluster 1:1st Floor (Office or Lobby) Release, Supply Fan OFF—1st Floor Log scale.

**Fig. 11:**
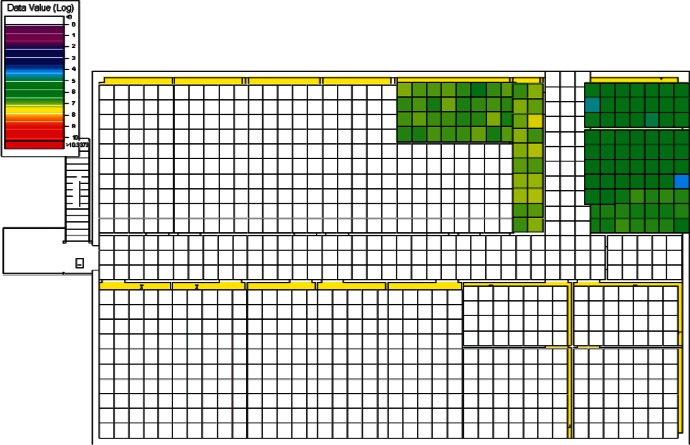
Cluster 1:1st Floor (Office or Lobby) Release, Supply Fan OFF—2nd Floor Log scale.

**Fig. 20:**
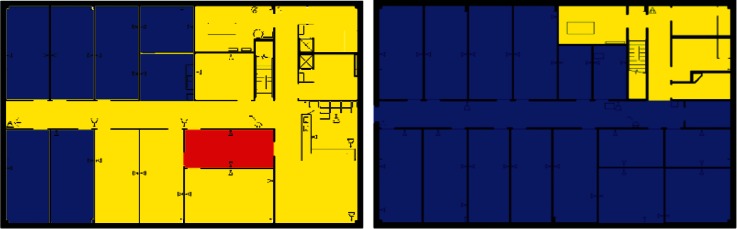
Cluster 1 Zoning strategy of 1st Floor and 2nd Floor for scenarios with 1st Floor Office Release and Supply Fans OFF (Red = Class 1, Yellow = Class 2, Blue = Class 3).

**Fig. 21:**
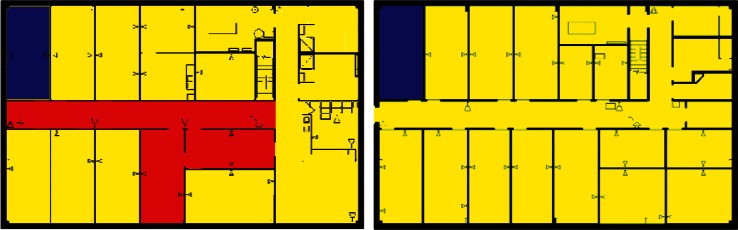
Cluster 2 Zoning strategy of 1st Floor and 2nd Floors for scenarios with 1st Floor Office Release and Supply Fans ON (Red = Class 1, Yellow = Class 2, Blue = Class 3).

**Fig. 22:**
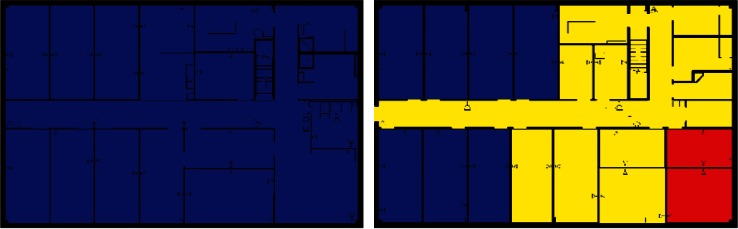
Cluster 3 Zoning strategy of 1st Floor and 2nd Floor for scenarios with 2nd Floor Office Release and Supply Fans OFF (Red = Class 1, Yellow = Class 2, Blue = Class 3).

**Fig. 23:**
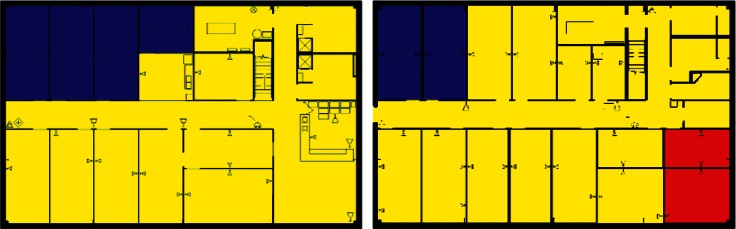
Cluster 4 Zoning strategy of 1st and 2nd Floor for scenarios with 2nd Floor Office Release and Supply Fans ON (Red = Class 1, Yellow = Class 2, Blue = Class 3).

**Fig. 24:**
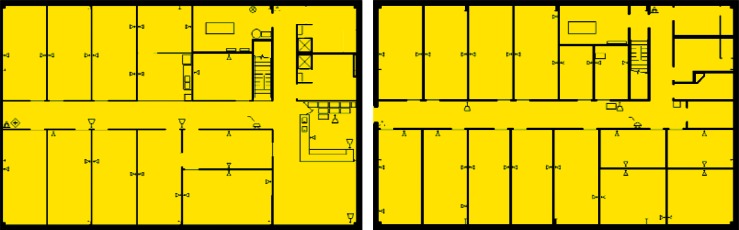
Cluster 5 Zoning strategy for 1st and 2nd Floor for scenarios with Outdoor Release (Yellow = Class 2).

The zoning strategy shown previously in Fig. 20 is designed for scenarios from Cluster 1 (1st Floor Releases with Supply Fan OFF). First, the four sampling strategies for this case will be applied by showing sample locations, summarizing the strategies in a table, and *overlaying* the sampling strategy onto the *ground truth data* based on the specific scenario shown in Fig. 10 and Fig. 11. The strategies will be compared to show how they differ with respect to the conclusions that can be drawn by applying them. Summaries and conclusions for the remaining four clusters will be provided in table form with respect to the zoning strategies applied on each floor.

Sample sizes for the purely judgmental and purely probabilistic approaches are the same for Clusters 2–5 as in Cluster 1. For the two strategies that include zoning, samples sizes within the zones are also the same in Clusters 2–5 as in Cluster 1, but it should be noted that some zoning strategies have fewer zones resulting in fewer samples. For instance, the 1st floor of Cluster 3 consists entirely of a Class 3 zone, so the conclusions are made without taking the additional samples needed for a separate Class 2 zone. Additionally, Cluster 5 has only Class 2 zones on both floors. Therefore, Cluster 5 scenarios do not need the additional samples needed for separate Class 1 and Class 3 zones.

It is important to note that the following comparisons of the four sampling strategies are based on single realizations of various sampling plans which have been applied to the data generated by CONTAM simulations which in this case are being taken as the ground truth. Consequently, the conclusions which can be drawn from a particular sampling design are relevant only for 1) the particular set of judgmental and/or probabilistic sample locations selected in that design and 2) the particular results generated by CONTAM for that simulation. For this reason, a rigorous statistical evaluation of the four sampling strategies would only be possible with thousands of realizations of the various sampling designs to verify the precision of their statistical confidence statements. Nonetheless, the application of the four sampling strategies to the five cluster scenarios below provides a valuable illustration of the strengths and weaknesses of each of the sampling strategies. A detailed report of a rigorous statistical validation of the sampling designs for buildings in VSP is currently in preparation (as part of the DHS funded Chemical Operation Technology Demonstration).

#### 4.4.1 Cluster 1—1st Floor Release With Supply Fan OFF

**Fig. 25:**
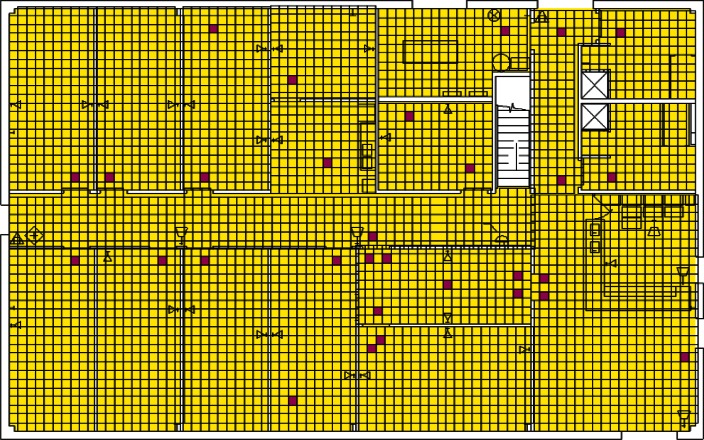
Purely Judgmental sample locations on 1st Floor.

**Fig. 26:**
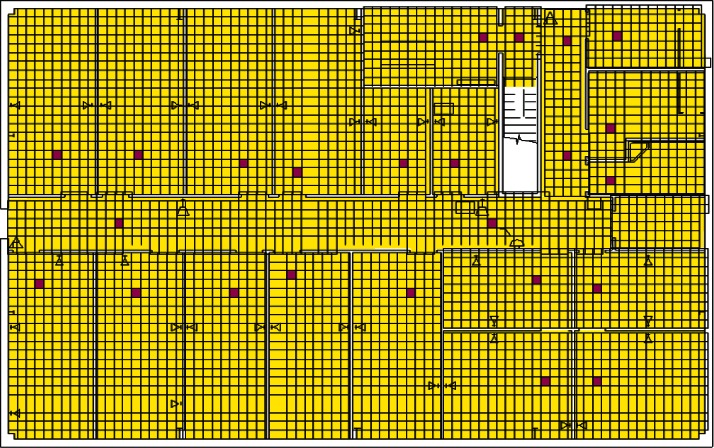
Purely Judgmental sample locations on 2nd Floor.

**Fig. 27:**
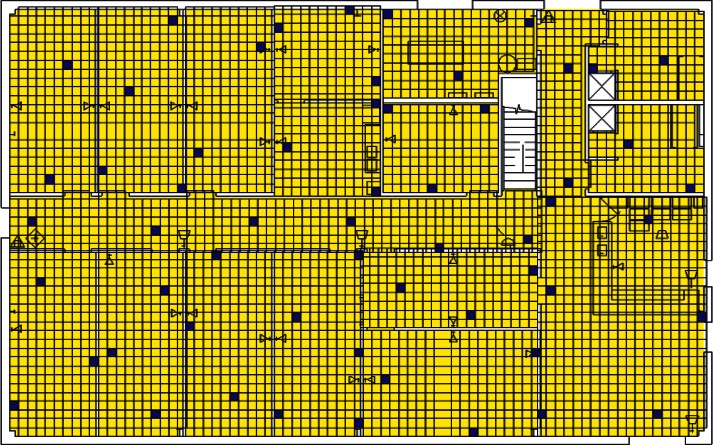
Purely Probabilistic sample locations on 1st Floor.

**Fig. 28:**
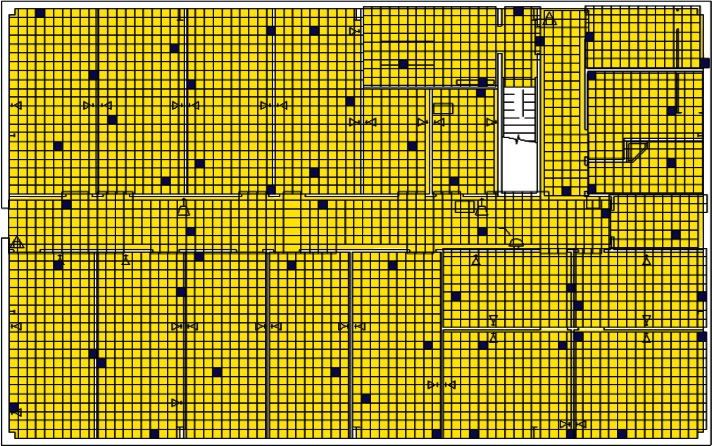
Purely Probabilistic sample locations on 2nd Floor.

**Fig. 29:**
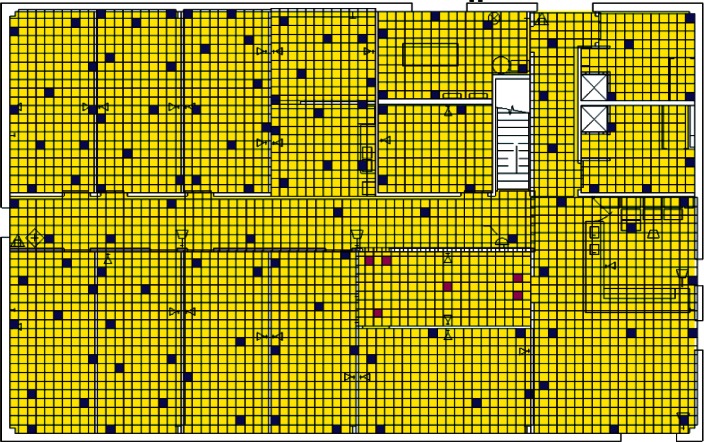
Purely Probabilistic with Zoning sample locations on 1st Floor. (Blue = random, ink = judgment).

**Fig. 30:**
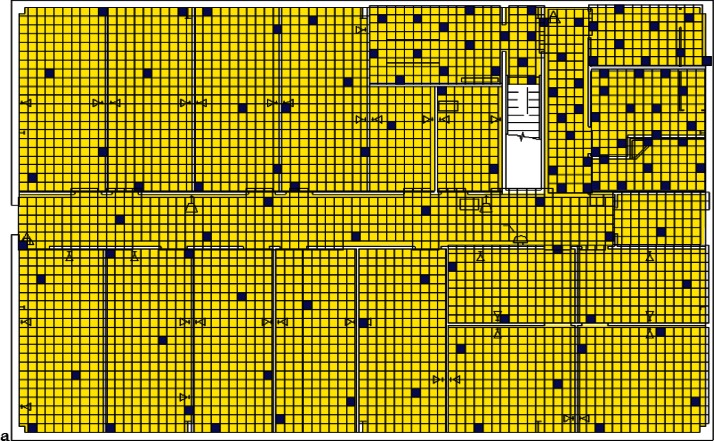
Purely Probabilistic with Zoning sample locations on 2nd Floor.

**Fig. 31:**
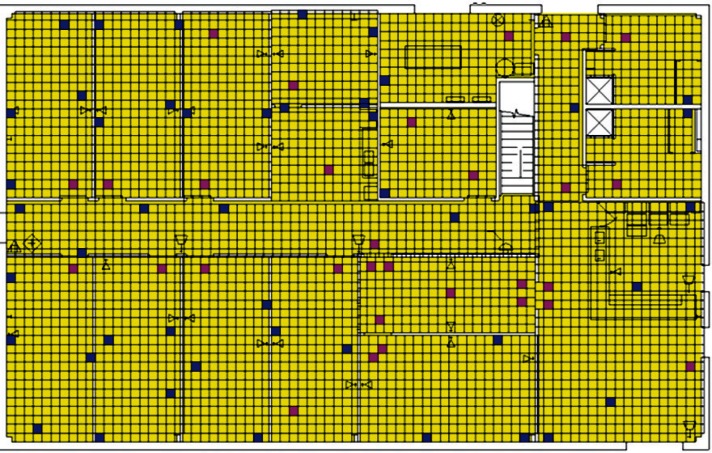
CJR with Zoning sample locations on 1st Floor. (Blue = random, Pink = judgment).

**Fig. 32:**
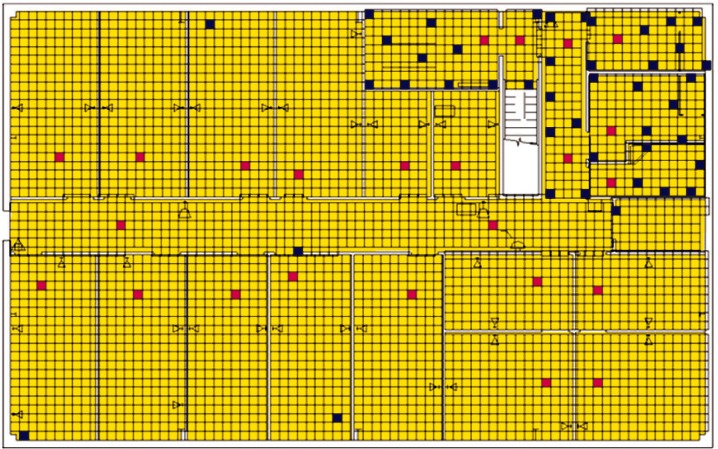
CJR with Zoning sample locations on 2ndFloor. (Blue = random, Pink = judgment).

Table 2 shows the four sampling methods and number of samples required for the Cluster 1 scenarios, i.e., 1st floor releases with the supply fans OFF. Sample locations for the purely judgmental approach are shown in Fig. 25 and Fig. 26. Sample locations for the purely probabilistic approach are shown in Fig. 27 and Fig. 28. Sample locations for the purely probabilistic with zoning approach are shown in Fig. 29 and Fig. 30. Sample locations for the CJR with zoning approach are shown in Fig. 31 and Fig. 32.

The *purely judgmental* approach proves useful in identifying some rooms as being contaminated. Based on the ground truth overlay (Fig. 10 and Fig. 11), many rooms were sampled once or twice with no samples exceeding the ACL level. This is not enough samples to state with a high degree of statistical confidence that the room is clean. Additional sampling would be required to confidently identify these rooms as clean. This shows that judgmental sampling is effective at identifying rooms as contaminated when all grid cells are contaminated.

The *purely probabilistic* approach also detected contaminated areas. However, because the two purely probabilistic sampling designs were applied to the entire 1st floor and the entire 2nd floor, the possible cleanliness of smaller areas on those floors could not be readily quantified with X %/Y % confidence statements. This is the case in the evaluation of the simulated scenario. If the samples are considered on an individual basis, many of the same conclusions drawn from the purely judgmental sampling approach can be made, i.e., some rooms can be identified as being contaminated. The purely probabilistic approach has the potential advantage of making X %/Y % confidence statements about an entire floor if none of the samples are above the ACL.

The *purely probabilistic* with zoning approach has a potential economic advantage over non-zoning approaches since the floors are sub-divided. This can lead to cost savings associated with eliminating portions of a floor as candidates for decontamination. When this sampling strategy is applied to the simulated scenario, the result is that the Class 3 zones on the 1st and 2nd floor are deemed 95 % confident that at least 95 % of the grid cells are below the ACL. This is more than half of the total surface area which could reduce decontamination costs if the building configuration and decontamination methods were amenable to such savings. It is important to note a 95 %/95 % was used in this exercise because of the tendency for rooms to be either entirely clean or entirely contaminated. In other types of release scenarios where there is the potential for very small portions of rooms to be contaminated, it is recommended that a stronger confidence statement be used such as 95 %/98 % or 95 %/99 %.

The CJR *with zoning* approach takes advantage of prior information to effectively make conclusions about some portions of the building while requiring fewer samples. The OR method yields similar conclusions to the purely probabilistic approach, but has the added advantages provided by both Bayesian prior and judgmental sampling. The Bayesian prior accounts for the probability that contamination is not present, which can be a reasonable assumption for Class 3 zones. This accounts for the sampling experts’ ability to identify likely areas of contamination, and results in much fewer samples as shown in Table 2, i.e., 145 for CJR with zoning versus 238 for purely probabilistic with zoning. It should be noted that the Bayesian prior used in this exercise was very conservative, and a less conservative (lower) Bayesian prior would result in fewer random samples being required.

#### 4.4.2 Cluster 2—1st Floor Release With Supply Fan ON

Conclusions from comparing the simulated scenario to the different sampling approaches for Cluster 2 are shown in Table 3. Because most rooms were contaminated, judgment sampling worked well at detecting contaminated areas. Purely probabilistic sampling also identified contaminated areas. However, because the purely probabilistic sampling design was applied to the entire 1st floor, the possible cleanliness of smaller areas on that floor could not be readily quantified with an X %/Y % confidence statement. Both of the sampling strategies using a zoning approach were able to categorize the Class 3 zones as uncontaminated with 95 % confidence that at least 95 % of the area was below the ACL, but the CJR with zoning approach required fewer samples.

#### 4.4.3 Cluster 3—2nd Floor Release With Supply Fan OFF

Conclusions from comparing the simulated scenario to the different sampling approaches for Cluster 3 are shown in Table 4. Judgment sampling worked well at detecting contaminated areas but was not able to clear the first floor as being uncontaminated due to insufficient sample size. Purely probabilistic sampling identified contaminated areas on the 2nd floor resulting in 95 % confidence that at least 95 % of the 1st floor was below the ACL. Both of the sampling strategies using a zoning approach were able to categorize the Class 3 zones (all of the 1st floor and part of the 2nd floor) as uncontaminated with 95 % confidence that at least 95 % of the area was below the ACL, but the CJR with zoning approach required fewer samples.

#### 4.4.4 Cluster 4—2nd Floor Release with Supply Fan ON

Conclusions from comparing the simulated scenario to the different sampling approaches for Cluster 4 are shown in Table 5. Because most rooms were contaminated, judgment sampling worked well at detecting contaminated areas. Purely probabilistic sampling also identified contaminated areas. However, because the two purely probabilistic sampling designs were applied to the entire 1st floor and the entire 2nd floor, the possible cleanliness of smaller areas on those floors could not be readily quantified with X %/Y % confidence statements. Both of the sampling strategies using a zoning approach were able to categorize the Class 3 zone on the 2nd floor as uncontaminated with 95 % confidence that at least 95 % of the area was below the ACL, but the CJR approach required fewer samples. Both of the strategies with zoning led to the reclassification of the 1st floor Class 3 zone as a Class 1 zone, because one of the rooms contained detectable contamination.

#### 4.4.5 Cluster 5—Outdoor Release

Conclusions from comparing the simulated scenario to the different sampling approaches for Cluster 5 are shown in Table 6. Because all of the rooms were contaminated on both floors, all of the methods conclude both floors are entirely contaminated.

#### 4.4.6 Summary of Sampling Strategy Comparisons

The scenarios examined have rooms which were either entirely clean or entirely contaminated. The number of samples for each method was sufficient enough so that each room was sampled at least once. This results in all sampling strategies being very effective at identifying the contaminated areas for these particular scenarios. Where the strategies differ is in the statements that can be made about areas where no contamination was found, and in the number of samples needed to make conclusions.

One key difference between the sampling strategies is their ability to make X %/Y % statements about areas where no contamination was found. The scenarios examined for Clusters 1 through 4 had rooms on at least one floor that were not contaminated. For each of these scenarios, the two strategies which used zoning were able to identify parts of the building where it was concluded with high confidence (95 %) that most (95 %) of these areas were uncontaminated, and for three of the four cases were able to identify such areas on both floors. The purely probabilistic strategy was able make an X %/Y % statement about the 1st floor of the scenario examined for Cluster 3. The purely judgmental strategy could not make an acceptable X %/Y % statement about any areas. In general, the methods using zoning were most effective at making X %/Y % statements and identifying areas which did not require decontamination. The CJR strategy with zoning required fewer samples while making the same conclusions as the purely probabilistic with zoning strategy.

Scenarios where an entire floor was contaminated were identified by all sampling strategies with the purely judgmental strategy requiring the fewest samples.

The results of this exercise support the expected strengths of the different methods. Purely judgmental sampling requires fewer samples for confirming contamination in areas known or highly likely to be contaminated. If areas can be classified as Class 3, applying a probabilistic or CJR sampling method allows the sampling team to make an X %/Y % about these areas if no contamination is found, and decontamination may not be required. When sampling experts have sufficient prior information about the release event, the CJR method allows X %/Y % statements to be made with fewer samples.

## 5. Conclusions

CONTAM and VSP have been shown to be useful for evaluating multiple sampling strategies as applied to buildings under a wide range of contaminant release, building configuration and operating scenarios. CONTAM can be used to perform virtual experiments to supplement actual experiments. Its strength lies in its ability to perform multiple simulations of a wide range of scenarios in relatively little time when compared to actual testing. VSP provides the capability to explore and employ a wide range of sampling strategies and has functionality specifically designed for the sampling of buildings. In combination, VSP and CONTAM provide a powerful set of tools with which investigators can study potential building-specific sampling strategies or obtain general knowledge related to building sample designs.

One of the fundamental outcomes of this project was the demonstration of how CONTAM and VSP can be used together to effectively develop sampling plans to support the various stages of response to a CBRN event. Following such an event, incident details and the conceptual site model could be used to create CON-TAM predictions of contaminant dispersion within a building. These predictions would then be used to identify priority area zones within the building and then sampling designs and strategies could be developed based on those zones.

Furthermore, CONTAM and VSP could potentially be used in preplanning exercises for a CBRN event. CONTAM and VSP can be used to explore a priori how one might best delineate priority sampling zones within a building in a manner applicable to a wide range of plausible scenarios. Viewing a large number of scenarios can allow the classification of an event based on a reduced set of parameters. This could allow sampling experts to initially choose better sampling approaches for different portions of the building, lead to clearing uncontaminated areas more quickly, provide a more accurate means of determining the number of samples required to meet specific sampling goals, and even improve targeting of decontamination efforts.

CONTAM and VSP can be used to evaluate the effectiveness of various sampling plans given varying ground truth scenarios. It was demonstrated that applying separate sampling designs within each zone can lead to some areas of the building being cleared when otherwise they would have been decontaminated or required further sampling. Together these tools can provide insight into the potential trade-offs between the number of samples, sampling locations, and associated uncertainty statements of the various sample designs that could be generated for a given set of scenarios.

CONTAM and VSP can be used to identify ground truth scenarios that yield similar contamination patterns, and thus could use the same sampling plan. Cluster analysis was used to show that contaminant dispersion patterns were similar across multiple scenarios, and therefore the same sampling plan could be used for such cases. This revealed the potential to reduce the number of sampling approaches or zoning schemes required to successfully cover a range of possible contamination scenarios.

This study was based on the uniform concentration assumption of CONTAM and empirically determined deposition rates from other studies. Consequently, hotspot scenarios were not addressed and variation in deposition due to other factors were not accounted for, e.g., surface roughness and orientation, airflow, temperature and humidity. CONTAM does provide the ability to simulate more detailed contaminant distribution on a sub-room scale, e.g., using one-dimensional convection/diffusion and computational fluid dynamics (CFD) within selected zones. This is another possible area to follow-up to utilize and improve upon the simulation of transport phenomenon within modeling tools in order to address a broader range of scenarios and to further evaluate the combined effectiveness of integrating sample planning tools.

The CONTAM-VSP tool could be used to leverage the integration of VSP with the Building Restoration Operations Optimization Model (BROOM) [23] developed by Sandia National Laboratories which is another DHS investment. An integrated CONTAM-VSP-BROOM tool would provide users with start-to-finish sample design and collection capability. CONTAM would provide simulated predictions to guide zoning strategies in order to create optimal sampling designs in VSP. These sampling designs would then be passed to BROOM to facilitate data acquisition and management. Data analysis of laboratory results could then be conducted by BROOM and VSP. Such a tool, when properly used, could reduce the time and cost of response and restoration by facilitating the rapid design and execution of validated, statistically defensible sampling plans.

## Figures and Tables

**Fig. 12:**
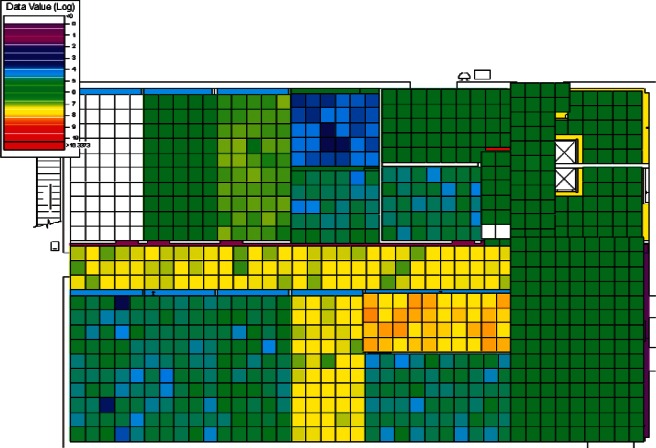
Cluster 2:1st Floor (Office or Lobby) Release, Supply Fan ON—1st Floor Log scale.

**Fig. 13:**
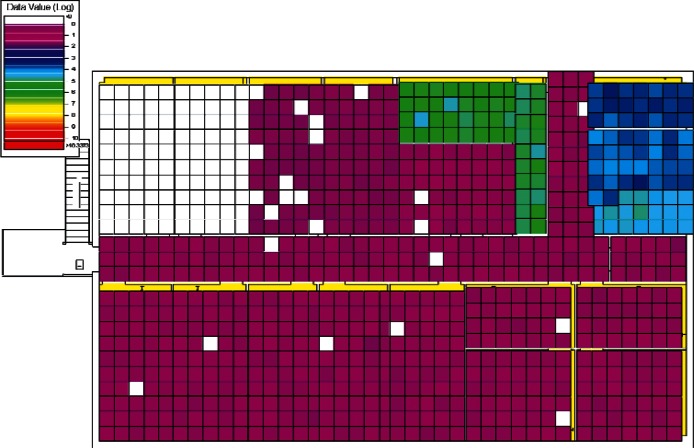
Cluster 2:1st Floor (Office or Lobby) Release, Supply Fan ON—2nd Floor Log scale.

**Fig. 14:**
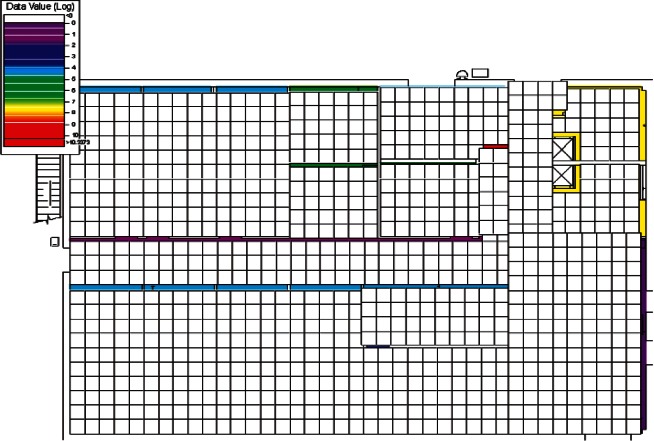
Cluster 3: 2nd Floor Office Release, Supply Fan Off—1st Floor Log scale.

**Fig. 15:**
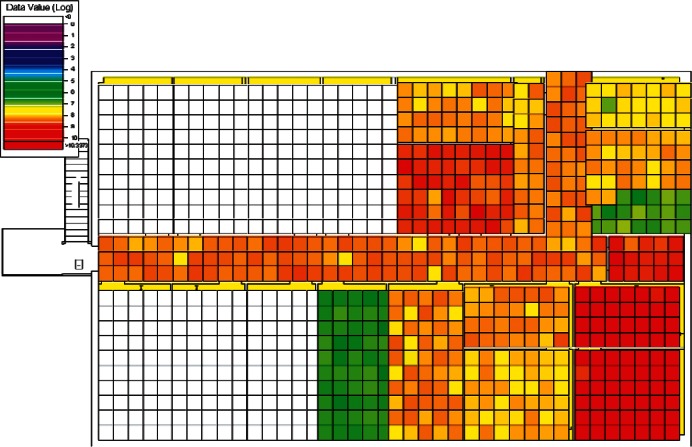
Cluster 3: 2nd Floor Office Release, Supply Fan Off—2ndFloor Log scale.

**Fig. 16:**
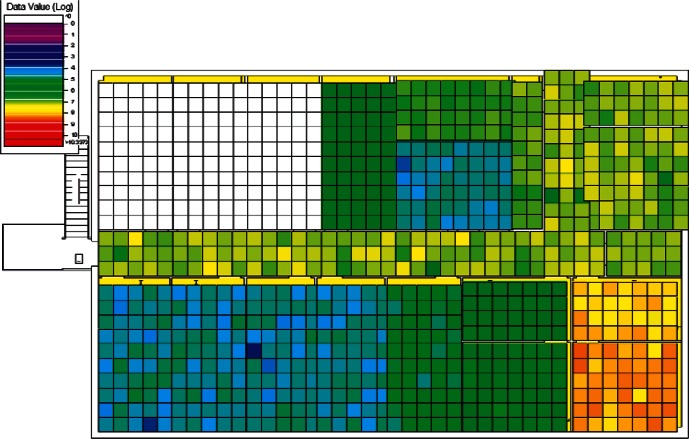
Cluster 4: 2nd Floor Office Release, Supply Fan ON—1st Floor Log scale.

**Fig. 17:**
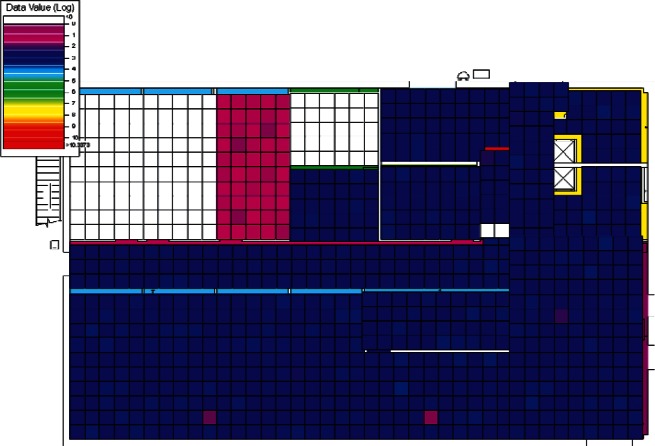
Cluster 4: 2nd Floor Office Release, Supply Fan ON—2nd Floor Log scale.

**Fig. 18:**
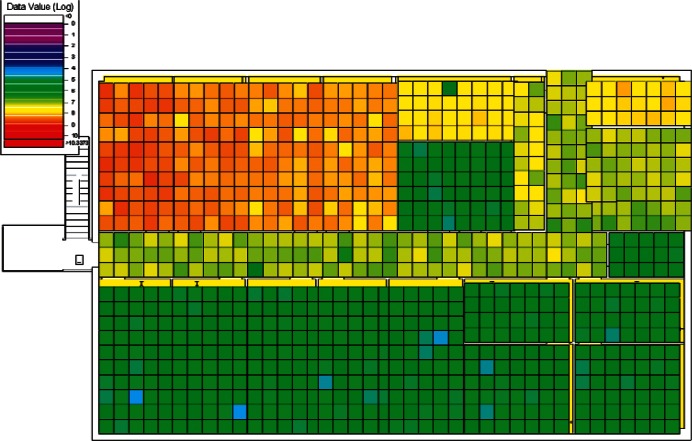
Cluster 5: Outdoor Release—1st Floor Log scale.

**Fig. 19:**
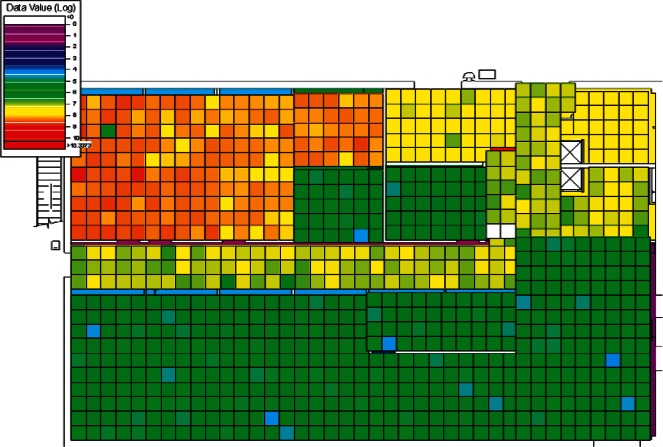
Cluster 5: Outdoor Release—2nd Floor Log scale.

**Table 1:** Factors and Levels for simulated scenarios

Factors	Levels	Number of Levels
**Contaminant Release**		
Location	Outdoor Remote 1st floor Lobby 1st floor Office 2nd floor Office	4
Amount	Low (1.0 mg) Medium (500 mg) High (1.0 g)	3
**HVAC Operation**		
1st and 2nd floor supply fans (and Outdoor air intake rate)	Both On (10 % outdoor air) Both Off (no outdoor air)	
1st and 2nd floor exhaust fans	On Off	2
Dedicated outdoor air fan	Inactive Active on 1st and 2nd floor offices	2
**Weather**		
Outdoor temperature	0°C 20°C	2
Wind speed	1 m/s 5 m/s	2
**TOTAL Simulations**		384

**Table 2:** Comparison of sampling strategies for Cluster 1(1st Floor Release with Supply Fans ON). Comparison based on scenario shown in Fig. 10 and Fig. 11

Sampling Strategy	Floor	Zone	#Judg. Samples	#Prob. Samples	Total	Conclusion if no samples above the Acceptable Contamination Level (ACL)	Comparison to Simulated Scenariao
Purely Judgmental	1	n/a	30	0	30	No contamination found, but cannot state statistical confidence	Several rooms contaminated, others possibly clean but no statistical confidence
	2	n/a	24	0	24	No contamination found, but cannot state statistical confidence	Several rooms contaminated, others possibly clean but no statistical confidence
**Total**			54	0	54		
Purely Probabilistic	1	n/a	0	58	58	95 % confident that 95 % of the floor is below the ACL	Some rooms contaminated
	2	n/a	0	58	58	95 % confident that 95 % of the floor is below the ACL	Some rooms contaminated
**Total**			0	116	116		
Purely Probabilistic with Zoning	1	Class 1	6	0	6	No contamination found, but cannot state statistical confidence	The room is contaminated
	1	Class 2	0	58	58	95 % confident that 95 % of the Zone is below the ACL	The rooms sampled are contaminated, and become class 1 zones.
	1	Class 3	0	58	58	95 % confident that 95 % of the floor is below the ACL	No contamination found. We are 95 % confident that at least 95 % of the zone is clean or below the ACL
	2	Class 2	0	58	58	95 % confident that 95 % of the floor is below the ACL	The rooms sampled are contaminated, and become Class 1 zones.
	2	Class 3	0	58	58	95 % confident that 95 % of the floor is below the ACL	No contamination found. We are 95 % confident that at least 95 % of the zone is clean or below the ACL
**Total**			6	232	238		
CJR with Zoning	1	Class 1	6	0	6	No contamination found, but cannot state statistical confidence	The room is contaminated
	1	Class 2*	16	24	40	95 % confident that 95 % of the floor is below the ACL	The rooms sampled are contaminated, and become class 1 zones.
	1	Class 3*	8	23	31	95 % confident that 95 % of the floor is below the ACL	No contamination found. We are 95 % confident that at least 95 % of the zone is clean or below the ACL
	2	Class 2*	7	39	46	95 % confident that 95 % of the floor is below the ACL	The rooms sampled are contaminated, and become class 1 zones.
	2	Class 3*	17	5	22	95 % confident that 95 % of the floor is below the ACL	No contamination found. We are 95 % confident that at least 95 % of the zone is clean or below the ACL
**Total**			54	91	145		

* Parameter inputs for CJR method: 50 % probability that judgment sample location contains detectable contamination and are twice as likely to contain detectable contamination as random sample locations.

**Table 3:** Comparison of sampling strategies for Cluster 2 (1st Floor Release with Supply Fans ON). Comparison based on scenario shown in Fig. 12 and Fig. 13

Sampling Approach	Floor	Class 1 Zones	Class 2 Zones	Class 3 Zones
Purely Judgmental AND Purely Probabilistic	1	All but one room contaminated. For this room, there is not a high level of confidence that the room is clean due to insufficient sample size.		
	2	All but two rooms contaminated. For these rooms, there is not a high level of confidence that the room is clean due to insufficient sample size.		
Purely Probabilistic with Zoning	1	Conclude the zone is contaminated	Conclude the zone is contaminated	Conclude with 95 % confidence that at least 95 % of the zone does not contain detectable contamination
	2	N/A		
CJR with Zoning	1	Conclude the zone is contaminated		Conclude with 95 % confidence that at least 95 % of the zone does not contain detectable contamination, but with fewer samples than purely probabilistic.
	2	N/A		

**Table 4:** Comparison of sampling strategies for Cluster 3 (2nd Floor Release with Supply Fans OFF). Comparison based on scenario shown in Fig. 14 and Fig. 15

Sampling Approach	Floor	Class 1 Zones	Class 2 Zones	Class 3 Zones
Purely Judgmental	1	No contamination was found, but *a statistical confidence statement cannot be made* due insufficient sample size and non-random sampling.		
	2	Contamination was found in slightly more than half of the rooms. For the uncontaminated rooms, there is not a high level of confidence that the rooms are clean due to insufficient sample size.		
Purely Probabilistic	1	No contamination was found. There is 95 % confidence that at least 95 % of the floor does not contain detectable contamination.		
	2	Contamination was found in slightly more than half of the rooms, thus it is concluded the floor contains detectable contamination. For several uncontaminated rooms, there is not high level of confidence that the rooms are clean due to insufficient sample size.		
Purely Probabilistic with Zoning	1	N/A		Conclude with 95 % confidence that at least 95 % of the floor does not contain detectable contamination.
	2	Conclude the zone is contaminated		Conclude with 95 % confidence that at least 95 % of the zone does not contain detectable contamination.
CJR with Zoning	1	N/A		Conclude with 95 % confidence that at least 95 % of the floor does not contain detectable contamination, but with fewer samples than purely probabilistic.
	2	Conclude the zone is contaminated		Conclude with 95 % confidence that at least 95 % of the zone does not contain detectable contamination, but with fewer samples than purely probabilistic.

**Table 5:** Comparison of sampling strategies for Cluster 4 (2nd Floor Release with Supply Fans ON). Comparison based on scenario shown in Fig. 16 and Fig. 17

Sampling Approach	Floor	Class 1 Zones		Class 2 Zones	Class 3 Zones	
Purely	1	Most rooms are contaminated. For three rooms not contaminated, there is not a high level of confidence that the room is clean due to insufficient sample size.				
Judgmental	2					

Purely Probabilistic	1	Contamination was found in most, and thus it is concluded the floor contains detectable contamination. For three uncontaminated rooms, there is not a high level of confidence that the rooms are clean due to insufficient sample size.				
	2					

Purely Probabilistic with Zoning AND CJR with Zoning	1	N/A	Conclude the zone is contaminated			Low levels of contamination were found in one room in the zone, and therefore the zone is reclassified as Class 1.
	
	2	Conclude the zone is contaminated				Conclude with 95 % confidence that at least 95 % of the zone does not contain detectable contamination, but with fewer samples than purely probabilistic.

**Table 6:** Comparison of sampling strategies for Cluster 5 (Outdoor Release). Comparison based on scenario shown in Fig. 18 and Fig. 19

Sampling Approach	Floor	Class 1 Zones	Class 2 Zones	Class 3 Zones
Purely Judgmental AND Purely Probabilistic	1	Conclude the entire floor is contaminated. All samples were contaminated.		
	2			
Purely Probabilistic with Zoning AND CJR with Zoning	1	N/A	Conclude the entire floor is contaminated. All samples were contaminated.	N/A
	2			
